# Dietary resveratrol increases the expression of hepatic 7α-hydroxylase and ameliorates hypercholesterolemia in high-fat fed C57BL/6J mice

**DOI:** 10.1186/1476-511X-11-56

**Published:** 2012-05-20

**Authors:** Qiong Chen, Ermao Wang, Liping Ma, Pei Zhai

**Affiliations:** 1 Department of food science, Guangdong Food and Drug Vocational College, Guangzhou, 510635, China

**Keywords:** Resveratrol, High-fat diet, LXRα, Bile acid pool size, Mice

## Abstract

**Background:**

Resveratrol (RSV), a naturally occurring polyphenolic stilbenoid, is known to possess potent anti-atherogenic properties; however, the effect of RSV on hypercholesterolemia is not fully understood. We hypothesized that RSV decreases blood cholesterol levels through the activation of cholesterol 7α-hydroxylase (CYP7A1)-mediated bile acid synthetic pathway pathways *in vitro* and *in vivo*.

**Methods:**

In this study, we evaluated body weight, serum lipid concentrations, hepatic lipid content and the size of the bile acid pool in high-fat diet (HFD)-fed C57BL/6 J mice that were treated with RSV. In addition, we characterized the underlying mechanism of the effects of RSV in HepG2 hepatocytes by Western blot analysis.

**Results:**

RSV (200 mg/kg per day) reduced body weight and liver weight gains, improved serum lipid parameters, reduced hepatic cholesterol accumulation and increased the bile acid pool size in mice fed an HFD for 8 wks. RSV significantly increased liver expression of CYP7A1 mRNA and protein and CYP7A1 enzyme activity. Furthermore, RSV treatment upregulated CYP7A1 expression and induced liver X receptor alpha (LXRα) activation in a time- and dose-dependent manner in HepG2 cells. In addition, the specific liver X receptor alpha (LXRα) inhibitor geranylgeranyl pyrophosphate (GGPP) inhibited the RSV-induced expression of CYP7A1 in HepG2 hepatocytes.

**Conclusion:**

The beneficial effects of RSV on HFD-induced hypercholesterolemia are mediated through LXRα signaling pathways, suggesting a potential target for the prevention of dyslipidemia.

## Background

Cardiovascular disease (CVD) is a major cause of morbidity and mortality in the United States [1]. It is widely accepted that hypercholesterolemia, especially an increase in the serum concentration of low-density lipoprotein cholesterol (LDL-C), is a major risk factor for atherosclerosis [2,3]. Reductions in LDL-C have been demonstrated to decrease CVD-related morbidity and mortality, and strategies aimed at lowering LDL-C remain a primary approach for cardiovascular risk reduction [4]. High-density lipoprotein cholesterol (HDL-C) has been shown to be inversely associated with the risk of CVD and thus is considered an anti-atherogenic lipoprotein. HDL-C exerts its anti-atherogenic properties primarily by facilitating the efflux of cholesterol from peripheral tissues and transporting it back to the liver by a process called reverse cholesterol transport.

Cholesterol 7α-hydroxylase (CYP7A1) catalyzes the first and rate-limiting step in the classical bile acid synthetic pathway in the liver [5]. The enzyme converts cholesterol into 7α-hydroxycholesterol, and subsequent enzymatic steps lead to the conversion of 7α-hydroxycholesterol into primarily cholic acid [6]. Because bile acid synthesis is the major pathway responsible for the maintenance of whole body cholesterol homeostasis, the regulation of CYP7A1 activity is important. Genetic deficiencies of CYP7A1 in humans are associated with hypercholesterolemia and the accumulation of cholesterol in liver [7].

Considerable attention has been focused on natural phytochemicals that may be beneficial in the prevention and treatment of CVD. Although drugs have been used as therapeutic regimens for lipid-related chronic diseases, there is little evidence that food factors themselves are directly beneficial in modulating lipid metabolism.

Resveratrol (3,5,4′-trihydroxy-trans-stilbene, RSV), a naturally occurring polyphenol, is widely present in a variety of plant species, including white hellebore (*Veratrum grandiflorum* O. Loes), *Polygonum cuspidatum*, grapes, peanuts and mulberries [8-10]. It is the major polyphenol found in red wine and it has been proposed to be the basis of the lower incidence of myocardial infarction in France than in comparable countries; the so-called “French paradox” [8-10]. Interest in resveratrol has increased due to its pharmacological effects, including cardio- and neuro-protection, and several other beneficial actions, e.g., anti-oxidant, anti-inflammatory, anti-carcinogenic and anti-aging. Despite the therapeutic effects of RSV, its impact on hypercholesterolemia remains unknown.

Because hepatic CYP7A1 plays an important role in the regulation of whole body cholesterol metabolism, we hypothesize that RSV improves hypercholesterolemia, inhibits hepatic cholesterol accumulation and increases bile acid synthesis via the activation of CYP7A1 signaling pathways *in vitro* and *in vivo*. To test our hypothesis, we determined the effects of RSV on serum and hepatic cholesterol accumulation in HFD-fed C57BL/6 J mice. In addition, we characterized the molecular mechanisms underlying the effects of RSV in HepG2 hepatocytes.

## Results

### Effects of RSV on body weight and food intake in HFD-fed mice

To investigate the effects of RSV on hypercholesterolemia, we used C57BL/6 J mice that were fed a HFD or RSV-supplemented HFD for 8 wks. Compared with ND-fed mice, the body and liver weights increased 14% and 11%, respectively, in HFD-fed mice. RSV reduced the body weight and the liver weight in HFD-fed mice (Table 1). The food intake did not significantly differ among the 3 groups throughout the 8-wk feeding period (data not shown). These results suggested that the beneficial effects of RSV on body and liver weights were not due to the lower levels of food intake.

**Table 1:** Body weight, food intake, and relative tissue weights in C57BL/6 J mice fed the ND, HFD or HFD + RSV diet for 8 wks

	**ND**	**HFD**	**HFD + RSV**
Initial body weight, g	20.5 ± 0.4	20.8 ± 0.4	21.2 ± 0.3
Final body weight, g	25.7 ± 1.5	31.8 ± 1.1	28.6 ± 1.7^*^
Food intake, g/d	3.45 ± 0.22	3.59 ± 0.41	3.48 ± 0.36
Liver, g/100 g body weight	4.67 ± 0.22	5.43 ± 0.13	4.97 ± 0.35^*^

Values are means ± SD. *n* = 10. Asterisks, significantly different from HFD (^*^P < 0.05).

### Effects of RSV on serum lipid concentrations in HFD-fed mice

Serum total cholesterol and LDL-C concentrations were significantly higher, whereas HDL-C concentrations were lower in HFD-fed mice compared with ND-fed mice (all *P* < 0.01). RSV-fed mice had lower total cholesterols and LDL-C, higher HDL-C levels compared with HFD-fed mice. Furthermore, the RSV group had a lower LDL-C/HDL-C ratio compared with the HFD group (Table 2). Triglyceride concentrations did not significantly differ among the 3 groups (Table 2).

**Table 2:** Serum lipid concentrations in mice fed ND, HFD or HFD + RSV for 8 wks

**Variables**	**ND**	**HFD**	**HFD + RSV**
Total cholesterol, mM	2.62 ± 0.16	4.17 ± 0.22	3.47 ± 0.19^**^
Triglyceride, mM	6.53 ± 0.32	5.96 ± 0.40	5.78 ± 0.35
LDL-C, mM	1.42 ± 0.19	2.94 ± 0.39	2.06 ± 0.20^**^
HDL-C, mM	1.12 ± 0.11	0.86 ± 0.10	1.11 ± 0.08^**^
LDL-C/HDL-C	1.28 ± 0.21	3.46 ± 0.67	1.87 ± 0.21^*^

^1^Results shown are means ± SD, *n* = 10. Asterisks, significantly different from HFD (^*^P < 0.05, ^**^P < 0.01).

### Effects of RSV on hepatic cholesterol contents in HFD-fed mice

The liver cholesterol content of from HFD-fed mice was higher than in the ND-fed mice. The hepatic accumulation of cholesterol in the RSV-fed mice was reduced by 46% compared with the mice fed the HFD (*P* < 0.05) (Table 3).

**Table 3:** Cholesterol concentrations and bile acid pool size in mice fed ND, HFD and HFD + RSV for 8 wks

	**ND**	**HFD**	**HFD + RSV**
Cholesterol, mg/g liver	48.9 ± 5.43	104.32 ± 8.92	56.37 ± 5.64^*^
Bile acid pool size, μM/g bw	1.79 ± 0.22	1.23 ± 0.15	2.46 ± 0.28^*^

Values are means ± SD, *n* = 10. Asterisks, significantly different from HFD (^*^P < 0.05).

### Effects of RSV on bile acid pool size in HFD-fed mice

Bile acid concentrations were lower in the HFD-fed mice than in the ND-fed mice (Table 3). The bile acid pool size of mice was 2-fold higher in the RSV-fed mice compared with mice fed the HFD diet (*P* < 0.05).

### Effects of RSV on hepatic CYP7α1 expression in HFD-fed mice

**Figure 1:**
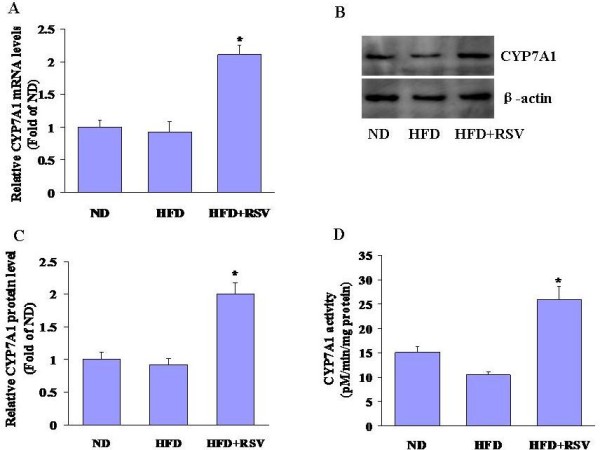
**RSV increases CYP7A1 expression and activity in HFD-fed mice.** After 8 wks of feeding, the liver microsomes of mice were isolated. (A) The CYP7a1 mRNA and (B) protein abundance were quantified as described in the Materials and Methods section. (C) Quantification of Western blot data. ^*^P < 0.05, *n* = 3. (D) CYP7A1 activity levels were expressed as pM/min/mg protein. Values are means ± SD, *n* = 6. Asterisks, significantly different from HFD-fed mice ( *P* < 0.05).

At the end of treatment, mice from each group were killed and liver CYP7α1 mRNA and protein expression were examined by quantitative RT-PCR and Western blot. CYP7α1 mRNA (Figure 1A) and protein (Figure 1B and 1C) levels in liver from RSV-treated mice were significantly elevated compared with the ND and HFD-fed mice. The RSV-fed mice showed a 2.5-fold increase in CYP7α1 enzyme activity compared with HFD-fed mice (Figure 1D).

### Effects of RSV on CYP7α1 expression in HepG2 hepatocytes

**Figure 2:**
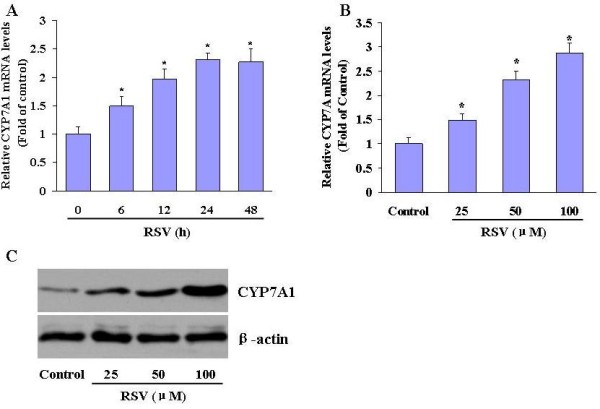
**RSV upregulates CYP7A1 expression in HepG2 cells.** (A) Time-dependent induction of CYP7A1 mRNA expression. Cultured HepG2 cells were incubated with RSV (50 μM) for the indicated times. CYP7A1 expression was then evaluated by quantitative RT-PCR. The figure shown is representative of 3 independent experiments. The abundance of CYP7A1 mRNA in untreated control cells was defined as 1 and the amounts of CYP7A1 mRNA from RSV-treated cells were then expressed as fold-changes in this control value. Asterisks, significantly different from 0 h ( *P* < 0.05). (B) Dose-dependent induction of CYP7A1 mRNA expression. HepG2 cells were treated with RSV for 24 h at the indicated concentrations and total RNA was isolated for the analysis of CYP7A1 and GAPDH mRNA expression by quantitative PCR. Asterisks, significantly different from control ( *P* < 0.05). (C) CYP7A1 protein expression was measured by Western blot.

To evaluate the effects of RSV on CYP7α1 expression, we exposed HepG2 cells to RSV (25, 50 and 100 μM) for different times and determined CYP7α1 expression. Resveratrol treatment was associated with a time-dependent increase in the expression of CYP7α1 mRNA, as determined by quantitative RT-PCR (Figure 2A). The levels of CYP7α1 mRNA gradually increased and were 2.3-fold higher at 24 h. The RSV treatment also increased CYP7α1 mRNA in a concentration-dependent manner (Figure 2B). Western blot analysis showed that RSV induces CYP7α1 protein expression in a dose-dependent fashion (Figure 2C).

### Effects of RSV on LXRα activation in HepG2 hepatocytes

**Figure 3:**
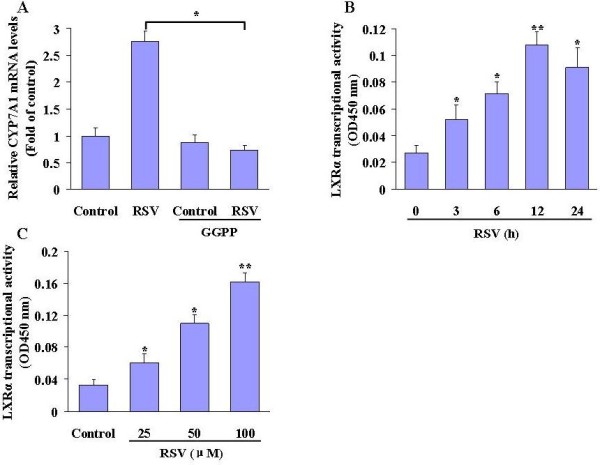
**LXRα mediates RSV-induced CYP7A1 expression in HepG2 cells.** (A) HepG2 cells were pre-incubated with vehicle or GGPP (10 μM) for 2 h, followed by RSV (50 μM) for an additional 24 h. Cellular lysates were subjected to quantitative RT-PCR to determine the mRNA levels of CYP7A1. (B) HepG2 cells were incubated with RSV (50 μM) for different times. (C) HepG2 cells were incubated with RSV (0, 25, 50 and 100 μM) for 12 h. The nuclear protein lysates were isolated and subjected to ELISA-based assay to determine the LXR transcriptional activity. The results are represented as the means ± SD of 3 individual experiments. Asterisks, significantly different from 0 hour or control ( *P* < 0.05).

The nuclear receptor liver X receptor alpha (LXRα) is a positive regulator of CYP7α1 transcription. To identify a potential mechanism of the effect of RSV on CYP7α1 expression, we used the LXRα inhibitor GGPP. The induction of CYP7α1 mRNA expression by RSV was largely prevented in the presence of GGPP [11]. Treatment with GGPP (10 μM) largely abolished the activity of RSV on CYP7α1 mRNA expression (Figure 3A). To determine whether RSV directly activates the LXRα pathway, we treated cells with RSV for different times and determined LXRα activation in control and RSV-treated cells. RSV treatment rapidly increased LXRα transcriptional activity and the kinetics of LXRα activation preceded the upregulation of CYP7α1 expression by RSV (Figure 3B). The activation of LXRα by RSV is also dose dependent (Figure 3C). These data indicate that activation of the LXRα pathway is necessary for RSV-induced expression of the CYP7α1 transcription.

## Discussion

Epidemiological studies suggest that the consumption of red wine may reduce the risk of cardiovascular disease. The cardio-protective effect of red wine has been attributed to the polyphenols present in red wine, in particular, RSV [12,13]. Pharmacological activities of RSV relevant to its putative cardio-protective and anti-atherogenic effects include reductions in platelet aggregation, LDL-C oxidation, and prostaglandin synthesis, as well as the promotion of endothelial nitric oxide synthase expression and activity [14,15]. However, the protective effects of RSV on dyslipidemia are not completely understood. In this study, we uncovered a novel mechanism for RSV in the amelioration of hypercholesterolemia. First, dietary RSV resulted in a significant reduction in total cholesterol and LDL-C, but an increase in HDL-C concentrations in HFD-fed C57BL/6 J mice. Second, supplementation with RSV decreased cholesterol accumulation and increased the bile acid pool size in the livers of HFD-fed mice. Third, RSV supplementation induced CYP7α1 mRNA and protein expression, as well as enzyme activity, in HFD-fed mice. Finally, RSV treatment increased CYP7α1 expression via activation of a nuclear receptor, LXRα, in HepG2 cells.

 C57BL/6 J mice have been identified as susceptible to the development of dyslipidemia on a diet containing cholesterol and high fat [16,17]. Consistent with former findings, the present study showed that after 8 wks of HFD feeding, C57BL/6 mice exhibited higher serum cholesterol and LDL-C levels, lower HDL-C levels, and greater accumulations of cholesterol in the liver. Supplementation of the HFD with RSV significantly lowered serum total cholesterol and LDL-C concentration compared with the HFD group. These findings strongly suggest that RSV is a promising new hypolipidemic compound. It is also noteworthy that RSV intake significantly increased HDL-C levels in HFD-fed mice. Elevated serum HDL-C has been significantly associated with a reduced risk of CVD by promoting transportation of cholesterol or cholesterol esters from peripheral tissues to the liver, where cholesterol is metabolized into bile acids [18,19]. This pathway represents a critically important mechanism for reducing cholesterol concentration in both the blood and peripheral tissues.

The liver is the only organ that is capable of degrading cholesterol whose degradation to bile acids occurs via “classic” (or neutral) and “alternative” (or acidic) bile acid biosynthetic pathways. The classic or neutral pathway, which is common to all mammals, is initiated by CYP7A1, an enzyme located in the endoplasmic reticulum; this enzyme catalyzes the initial step in the pathway of bile acid synthesis, which is responsible for more than 50% of bile acid formation [20-22]. Deficiency in CYP7A1 manifests with markedly elevated total cholesterol and LDL-C, premature gallstones, and premature coronary and peripheral vascular disease. In this study, we found that CYP7A1 mRNA and protein abundance was significantly decreased in HFD-fed mice compared with ND-fed mice. Supplementation of the HFD with RSV caused a significant induction of CYP7A1 mRNA and protein expression compared with the HFD alone. Liver CYP7A1 activity was also enhanced in RSV-fed mice compared with HFD-fed mice. As a result, hepatic cholesterol levels were reduced and the bile acid pool size was increased in RSV-fed mice compared with HFD-fed mice.

Liver X receptor α (LXRα), a nuclear hormone receptor, has been implicated in the feed-forward and feedback regulations of CYP7A1 [23]. LXRα heterodimerizes with the retinoid X receptor (RXR) and is activated by specific cholesterol derivatives called oxysterols. *In vitro* studies have shown that ligand-bound LXRα mediates transcriptional up-regulation of CYP7A1 by binding to an LXR regulatory element in the CYP7A1 promoter [24]. Physiological evidence for this process has been provided by studies using LXRα knock-out mice (LXR^−/−^), which fail to up-regulate CYP7A1 in response to cholesterol feeding, and as a result accumulate large amounts of cholesterol in their livers [25]. To further examine the molecular mechanism by which RSV regulates CYP7A1 expression, we analyzed the impact of RSV on LXRα. Treatment of HepG2 cells with RSV dose-dependently increased LXRα transcriptional activity, indicating LXRα activation. Inhibition of LXRα activity by the inhibitor GGPP abrogated the RSV-mediated upregulation of CYP7A1. These results suggest that LXRα-mediated transcriptional regulation is required for the induction of CYP7A1 by RSV.

In summary, the present study demonstrated that dietary RSV increased CYP7A1 expression and bile acid pool size, resulting in decreased blood cholesterol and LDL-C concentrations.

## Methods

### Animals and diets

C57BL/6 mice were obtained from Jackson Laboratories (Bar Harbor, ME, U.S.A.). The normal diet (ND) and the high fat diet (HFD, 45% kcal fat; D12451) (Table 4) were obtained from Research Diets, Inc. (New Brunswick, NJ, USA). All the animals were maintained in a 22°C room with a 12-h light/dark cycle and received drinking water ad libitum. The mice were fed the ND to acclimate to the environment for 2 wks prior to the start of experiment. Then, thirty eight-week-old male C57BL/6 J mice were randomly divided into 3 groups of ten mice per group and fed the ND, the HFD or the HFD supplemented with RSV (200 mg/kg diet per day). The experiment lasted 8 wks, and the body weights and food intake were recorded weekly. At the end of the experimental period, all animals were anesthetized with ether, and blood was collected by heart puncture. The livers were excised, weighed, and further examined. All animal procedures were in accordance with the approved protocol for the use of experimental animals determined by the standing committee on animal care at Guangdong Food and Drug Vocational College.

**Table 4:** Composition of ND and HFD diets

	**ND (g/kg diet)**	**HFD (g/kg diet)**
Ingredient (g/kg diet)		
Casein	200	200
Sucrose	500	172.8
Lard	0	177.5
Soybean oil	50	25
Starch	150	72.8
Cellulose	50	50
Mineral mix^1^	35	35
Vitamin mix^1^	10	10
L-cysteine	3	3
Choline bitartrate	2	2
RSV	0	0
Energy (kJ/g)	0.88	19.87
Protein (% kJ/kg)	13.3	20
Fat (% kJ/kg)	8.0	45

Forty-five percent kilojoules per fat pellet diets (D12451; Research Diet) were used as HFD, and normal chow diet were used as ND.

### Cell culture

HepG2 cells, a human hepatoma cell line, were cultured in Dulbecco's modified Eagle's medium (DMEM) supplemented with 10% fetal bovine serum (FBS) and antibiotics at 37°C in a humidified, 5% CO2/95% air atmosphere. The cells were incubated with RSV (Sigma-Aldrich) at various concentrations and for the indicated time periods.

### Serum lipid concentrations

Blood samples were obtained by retro-orbital bleeding under ether anesthesia. The serum was separated by centrifugation at 2000 × *g* for 10 min at 4°C and stored at −70°C before analysis. When thawed, the samples were centrifuged at 1800 ×  *g* for 10 min and diluted 1:10 in PBS/BSA (1%), pH 7.4. Enzymatic colorimetric methods on a Roche Hitachi 911 Chemistry Analyzer (Roche Diagnostics, Minnesota, USA) were used to measure total cholesterol, LDL-C, HDL-C and triglyceride (Wako Chemicals, Richmond, VA).

### Hepatic cholesterol levels

The liver was removed immediately and rinsed with cold PBS after the animal had been euthanized. The liver was dried, minced, and weighed before extracting the lipids by the method described by Folch et al. [26]. The TC content of the liver was determined by using an enzymatic fluorometric assay based on a modification of previously described methods [27]. The assays were performed in duplicate. All values are expressed as μM/g tissue per animal.

### Bile acid pool size

The pool size was defined as the bile acid content of the small intestine, liver, and gallbladder. These tissues were removed and homogenized in double distilled water at room temperature with a Polytron homogenizer (Kinematica, Kùens-Lucerne, Switzerland). The homogenate was then extracted in ethanol and the total bile acid content of the tissue extracts was measured with an enzymatic assay (Trinity Biotech, Bray, Ireland) [28].

### Quantitative RT-PCR

Liver (50 mg) was homogenized in 1 ml of TRIzol (Life Technologies, Gaithersburg, MD, U.S.A.), and total RNA was extracted following the manufacturer’s protocol. The RNA was quantified using spectrophotometric analysis at OD260. RNA integrity was checked by agarose gel electrophoresis. Oligo(deoxythymidine)-primed cDNA was synthesized from 0.5 μg RNA samples using the Promega Reverse Transcription System (Promega, Southampton, Hants, U.K.). Using the ABI PRISM 7900 Sequence Detection System (PE Applied Biosystems, Cheshire, U.K.), transcript level quantification was performed with commercially available real-time PCR primer-probe sets. GAPDH was used as the endogenous control. The relative amount of mRNA was determined from standard curves generated for both the target and the endogenous reference using serial dilutions of cDNA [29].

### Western blot

Protein extracts of the liver microsomes were prepared, and the protein concentration was determined using a Bio-Rad Protein Assay kit (Bio-Rad, Hercules, CA). Proteins were size-fractionated electrophoretically using sodium dodecyl sulfate-polyacrylamide gel electrophoresis (SDS-PAGE) gels and transferred to polyvinylidene fluoride membranes (PVDF, Millipore). The membranes were incubated with the primary anti-CYP7A1 antibody (Abcam), and HRP-labeled secondary antibody (Cell Signaling Technology Inc.) and were visualized using an enhanced chemiluminescence detection system (ECL, Roche). Anti-β-actin (Cell Signaling Technology Inc.) was used as the loading control.

### CYP7A1 activity

Liver samples were frozen immediately in liquid nitrogen. Microsomes were prepared by differential ultracentrifugation [30] and used for the measurement of CYP7A1 activity using an ELISA-based assay kit (R&D Systems).

### LXRα transcriptional activity assay

Nuclear extracts of cells were prepared as described previously [31]. Briefly, monolayers (2 × 10^6^ cells) were harvested by scraping, washed in cold PBS, and incubated in two packed cell volumes of buffer A (10 mM HEPES [pH 8.0], 1.5 mM MgCl_2_, 10 mM KCl, 0.5 mM dithiothreitol, 200 mM sucrose, 0.5 mM phenylmethylsulfonyl fluoride, 1 mg/ml leupeptin and aprotinin, and 0.5% Nonidet P-40) for 5 min at 4°C. The nuclei were collected by microcentrifugation, rinsed once in buffer A, and resuspended in two-thirds packed cell volume of buffer C (20 mM HEPES [pH 7.9], 1.5 mM MgCl_2_, 420 mM NaCl, 0.2 mM EDTA, 0.5 mM phenylmethylsulfonyl fluoride, 1.0 mM dithiothreitol, 1.0 μg/ml leupeptin and aprotinin). The nuclei were incubated at 4°C for 20 min and clarified by microcentrifugation for 5 min. The resulting supernatants were used as the nuclear fraction, and the protein concentration was determined by the Bradford method (26). LXRα transcription factor activity was assayed by an enzyme-linked immunosorbent assay-based LXRα transcription factor activity assay kit to detect and qualify transcription LXRα factor activation (Active Motif Inc.).

### Statistical analyses

The results are expressed as the means ± SD. The data were analyzed by one-way ANOVA coupled with Student–Newman–Keuls multiple comparison tests. Differences were considered significant if *P* < 0.05. SPSS 16.0 software was used for all statistical analyses.

## Abbreviations

CYP7A1, cholesterol 7α-hydroxylase; CVD, cardiovascular disease; DMEM, dulbecco modified eagle medium; GGPP, geranylgeranyl pyrophosphate; HDL-C, high density lipoprotein cholesterol; HFD, high-fat diet; LDL-C, low density lipoprotein cholesterol; LXRα, liver X receptor alpha; ND, Normal diet; RSV, Resveratrol.

## Competing interests

The authors declare that they have no competing interests.

## Authors’ contributions

QC and EW conceived the idea and designed the study. LM and PZ carried out the animal experiments. QC performed the statistical analysis and interpretation of the data. QC and EW conducted the cell experiments. QC and EW drafted the manuscript and provided critical corrections to the manuscript. All authors read and approved the final manuscript.
